# Caregiver reactions to neuroimaging evidence of covert consciousness in patients with severe brain injury: a qualitative interview study

**DOI:** 10.1186/s12910-021-00674-8

**Published:** 2021-07-28

**Authors:** Andrew Peterson, Fiona Webster, Laura Elizabeth Gonzalez-Lara, Sarah Munce, Adrian M. Owen, Charles Weijer

**Affiliations:** 1 Institute for Philosophy and Public Policy, George Mason University, Fairfax, USA; 2 Arthur Labatt Family School of Nursing, Western University, London, Canada; 3 Brain and Mind Institute, Western University, London, Canada; 4 Toronto Rehabilitation Institute-University Health Network, Toronoto, Canada; 5 Departments of Medicine, Epidemiology & Biostatistics, and Philosophy, Western University, London, Canada

**Keywords:** Vegetative state, Minimally conscious state, Neuroimaging, Caregiver, Ethics, Research ethics, Traumatic brain injury

## Abstract

**Background:**

Severe brain injury is a leading cause of death and disability. Diagnosis and prognostication are difficult, and errors occur often. Novel neuroimaging methods can improve diagnostic and prognostic accuracy, especially in patients with prolonged disorders of consciousness (PDoC). Yet it is currently unknown how family caregivers understand this information, raising ethical concerns that disclosure of neuroimaging results could result in therapeutic misconception or false hope.

**Methods:**

To examine these ethical concerns, we conducted semi-structured interviews with caregivers of patients with PDoC who were enrolled in a concurrent neuroimaging research program designed to detect covert consciousness following severe brain injury. Caregivers held surrogate decision-making status for a patient. Interviews were conducted at two time points for each caregiver. The first interview occurred before the disclosure of neuroimaging results. The second occurred after disclosure. Descriptive analysis was applied to the data of four interview topics: (1) expectations for neuroimaging; (2) reactions to evidence of preserved cognition; (3) reactions to null results; and (4) understanding of the results and study.

**Results:**

Twelve caregivers participated in the study; two caregivers shared surrogate decision-making status for one patient with PDoC. Twenty-one interviews were completed; one caregiver declined to participate in the post-disclosure interview. Three patients with PDoC associated with the study displayed evidence of covert consciousness. Overall, caregivers understood the neuroimaging research and results. Caregivers who received results of covert consciousness were generally pleased. However, there was some variation in expectations and reactions to these data and null results.

**Conclusion:**

This study, for the first time, reveals caregiver expectations for and reactions to neuroimaging evidence of covert consciousness in patients with PDoC. Caregivers understood the neuroimaging research and results, casting doubt on speculative ethical concerns regarding therapeutic misconception and false hope. However, disclosure of neuroimaging result could be improved. Pre-disclosure consultations might assist professionals in shaping caregiver expectations. Standardization of disclosure might also improve comprehension of the results.

**Supplementary Information:**

The online version contains supplementary material available at 10.1186/s12910-021-00674-8.

## Background

Brain injury is medically challenging and places significant burdens on family caregivers (hereafter, “caregivers”) and health systems. Severe brain injury can lead to prolonged disorders of consciousness (PDoC), such as the vegetative state (VS) and minimally conscious state (MCS). Diagnosis and prognostication of patients with PDoC is difficult. Misdiagnosis occurs often and caregivers frequently make ethically fraught decisions, such as whether to continue life-sustaining treatment, under conditions of uncertainty.

Recent advances in neurology might improve diagnosis and prognostication in some patients with PDoC. Researchers have developed functional magnetic resonance imaging (fMRI) and electroencephalography (EEG) methods that improve diagnostic and prognostic accuracy following severe brain injury [1–3]. Some studies demonstrate that brain responsiveness to auditory stimuli predicts recovery [4, 5]. Other studies demonstrate that some patients clinically diagnosed as being in a vegetative state can willfully modulate their brain activity to command, indicating that they are aware [6–9]. These patients are regarded as “covertly conscious” or as having “cognitive motor dissociation”— their consciousness is manifest in their brain activity, not their overt behavior, and neuroimaging is the only way to detect it [10, 11].

The evidence base for these methods is relatively nascent and they are still used predominantly in the research setting. However, several key developments in neurology practice guidelines have occurred over the past three years, signaling that fMRI and EEG assessment of patients with PDoC might soon be incorporated in clinical practice. The 2018 U.S. practice guideline update on disorders of consciousness recommends that such methods may be used for prognostication or if diagnosis with serial clinical examination remains ambiguous [12]. In 2020, the European Academy of Neurology made similar recommendations about the potential benefit of routine fMRI and EEG assessment in patients with PDoC [13].

Despite these advancements, disclosure of neuroimaging results of covert consciousness remains an area of intense ethical debate. In the research setting, ethicists worry that disclosure could lead to therapeutic misconception [14]. Fins and colleagues, for example, “caution against suggesting therapeutic intent when there is none and thus fostering a therapeutic misconception” [14, page 9]. They go on to stress that “actively disabusing surrogates of such misconceptions” should be a “an even stronger goal” of research programs using these methods. Similarly, ethicists also argue that, once these methods are translated into clinical practice, they could negatively impact decision making in the early phase of recovery. Caregivers could develop false hope in a positive outcome and might miss the “window of opportunity” to withdraw life-sustaining treatment (e.g., [15]). These arguments reflect a shared concern that, in both the research and clinical settings, caregivers of patients with PDoC might be emotionally vulnerable and misunderstand the results. Complex neuroimaging data could be difficult to process, leading caregivers to reason “beyond the evidence” and make poorly informed clinical decisions.

These arguments highlight important considerations for researchers and physicians. However, many of these claims are speculative. The fact that disclosure of neuroimaging evidence of covert consciousness could lead to therapeutic misconception or false hope does not mean that it will. Contrary to these cautionary claims, we have argued that neuroimaging results ought to be disclosed to caregivers, provided that four conditions are met [16]. First, disclosure should not undermine the scientific validity of the study. Second, the results must be informative and reliable. Third, the benefits of disclosure must outweigh potential harms. And fourth, caregivers must consent to disclosure. However, caregivers’ expectations for and experiences of disclosure—particularly in how they understand the benefits, harms, and informativeness of neuroimaging data—are still poorly understood.

In this article, we present findings from a qualitative interview study that assessed caregivers’ expectations for and experiences of multi-modal neuroimaging research to detect covert consciousness in patients with PDoC. One previous study has examined this issue [17], but the study focused on EEG assessment in the European context, and the retrospective design limited examination of participant attitudes [18]. By contrast, our study prospectively examined participant attitudes as caregivers progressed through the neuroimaging process.

Our study is part of a four-year research program on the ethics of neuroimaging following severe brain injury [19]. This program was, in part, guided by four research questions: 1) What are caregivers expectations for enrolling in neuroimaging research?; 2) How do caregivers react to evidence of covert consciousness?; 3) How do caregivers react to uninformative neuroimaging results?; and 4) Do caregivers generally understand the research and results? These research questions are informed by the ethical concerns of therapeutic misconception and false hope, outlined above. Empirical assessment of caregivers’ experiences might clarify these ethical debates, improve the disclosure of neuroimaging results, and optimize physician-caregiver communication as these methods are translated into clinical practice.

## Methods

### Research context

We recruited caregivers of patients with PDoC from a concurrent neuroimaging research program at the Owen Lab at the University of Western Ontario, an internationally recognized center for brain injury research. Patients assessed by the Owen Lab include individuals clinically diagnosed as being in the VS, MCS, or locked in syndrome (LIS). The VS is a neurological condition characterized by wakeful unresponsiveness [20, 21]. The MCS is characterized by wakefulness with intermittent behavioral responsiveness to visual, auditory, tactile, or noxious stimuli [22]. The LIS is characterized by whole body paralysis but preserved cognitive function [23]. Patients with these conditions have profound motor impairments, which can conceal preserved cognition during clinical examination. To verify clinical diagnosis, patients were repeatedly assessed with the JFK-Coma Recovery Scale-Revised [24] when they visited the lab.

Patients with PDoC whose caregivers were enrolled in our interview study underwent a variety of neuroimaging tests, including fMRI scans and EEG recordings (see Table 1). The neuroimaging tests were designed to detect preserved cognitive functions following brain injury—such as basic executive and auditory functions—or the capacity to volitionally modulate brain activity to command. A lab coordinator (LGL), with PhD training in brain injury, guided caregivers through the neuroimaging research. Neuroimaging results were disclosed to caregivers by the lab PI (AMO) and lab coordinator (LGL). The disclosure process included a results document and phone consultation, with opportunities for follow-up discussion. Identical results documents were sent to the referring physician and the caregiver.

**Table 1:** Selected neuroimaging studies in which patients were enrolled

Study	Modality	Purpose of study	Main outcome
Naci and Owen [25]	fMRI	To determine whether fMRI can detect selective attention command following and communication	Three patients with PDoC were capable of selective attention command following. Two of these patients also responded correctly to yes/no questions
Beukema et al. [4]	EEG	To determine whether event-related potentials can detect preserved auditory processing	All enrolled patients with PDoC (N = 16) showed preserved auditory processing. Seven showed differentiated speech from noise
Gibson et al. [26]	EEG and fMRI	To determine whether somatosensory functions can serve as proxies for covert consciousness as compared to fMRI or clinical evaluation	Only those patients with PDoC who were capable of fMRI or behavioral command following also demonstrated relevant somatosensory functions
Naci et al. (27)	fMRI	To determine whether fMRI can detect covert consciousness with naturalistic stimuli (e.g., viewing a movie)	A model of cognitive changes during movie viewing was developed in healthy participants. Cognitive changes in one patient with PDoC were identical to those in the model while viewing the movie

The results document contained descriptions of the neuroimaging tests, patient responses, and interpretations of the responses (see Additional file 1). The majority of the document described the results in lay terms, while portions were written in more technical language for referring physicians.

### Participants

Caregivers of patients with PDoC were sequentially recruited from the concurrent neuroimaging research at the Owen Lab. We interviewed caregivers who spoke English, were surrogate decision makers for the patient with PDoC, and who had cared for the patient for at least six months. Consistent with established qualitative methods [28], we recruited and interviewed participants until thematic saturation was reached, the point at which no new qualitative themes emerge from the interview data.

### Data collection

An interview guide was developed by our research team (LGL, AMO, AP, FW, CW) based on a literature review and expert knowledge of themes of interest (see Additional file 2: interview guide). Themes of interest included the history of the brain injury, reasons for enrolling in the neuroimaging research, reactions to evidence of preserved cognition or null results, changes in caregiver or clinician behavior, and the overall experience of the neuroimaging research program. Interviews were digitally recorded and professionally transcribed.

Semi-structured interviews were performed by an experienced qualitative researcher (SM). We conducted two interviews for each participant. The first interview occurred prior to the disclosure of neuroimaging results. The second occurred within two months after disclosure. The interview guide contained pre- and post-disclosure questions to assess changes in caregiver attitudes. The time intervals between interviews, neuroimaging, and disclosure were variable due to scheduling constraints and neuroimaging unit availability. However, no participant interview was conducted more than two months before neuroimaging or two months after the disclosure of results.

Data collection and analysis were iterative and concurrent. Four members (LGL, SM, FW, CW) of our interdisciplinary team independently read and coded transcripts before regularly scheduled discussions. The interview guide was then adapted to new insights and themes that emerged from each interview. Through dialogue, team members developed codes that described the meaning units of the qualitative data. Open coding was first applied so identified codes were descriptive and grounded in the data. A coding framework was then developed and applied across all transcripts using the software, NVivo-10. Axial coding was used to compare relationships between codes. For this article, a descriptive analysis was applied to the interview data related to our research questions: 1) caregiver expectations for neuroimaging; 2) caregiver reactions to neuroimaging evidence of preserved cognition; 3) caregiver reactions to null results; and 4) caregiver understanding of the results and study. The analysis was reviewed by four members of the research team for accuracy (LGL, AP, FW, CW).

## Results

Twelve caregivers (eight self-identified as female) of eleven patients with PDoC participated in the study (Table 2). Seven caregivers were parents, four were spouses, and one was a sibling. All caregivers were Canadian residents and interacted with provincial health systems when managing benefits and care for patients. Six patients with PDoC were clinically diagnosed as being in the VS (P1, P4, P6, P8, P10, P11), one was clinically diagnosed as being in the MCS (P2), three alternated between the VS, MCS, and emergence from the MCS (P3, P5, P9), and one was clinically diagnosed as being in the LIS (P7). The average time since injury was 5.49 years (SD = 7.14). The parents of one patient reported that his fiancée acted as the primary caregiver and surrogate decision maker after injury, but later relinquished this role to them. All other caregivers had been serving in this role since the patient’s initial injury.

**Table 2:** Caregiver and patient characteristics

Caregiver	Relation to patient	Clinical diagnosis of patient	Time since injury	Main neuroimaging results	Covert consciousness
P1	Spouse	VS	5 yrs	No significant findings with EEG or fMRI	No evidence
P2	Mother	MCS	4.1 yrs	EEG revealed basic attentional capacity. fMRI data had movement artifacts and no conclusions could be drawn	No evidence
P3	Father	VS/MCS	17 yrs	EEG revealed basic attentional capacity and speech versus noise distinction. fMRI revealed visual and executive functions, and selective attention command following*	+
P4	Spouse	VS	1.2 yr	No significant findings with EEG. No fMRI tests performed	No evidence
P5	Spouse	VS/MCS	3.1 yrs	EEG revealed speech from noise distinction. fMRI revealed capacity for selective attention command following*	+
P6	Mother	VS	22 yrs	EEG revealed attentional capacity. fMRI revealed capacity for mental imagery*, selective attention command following*, and communication*	+
P7	Spouse	LIS	1 yr	EEG revealed basic attentional capacity. No fMRI tests were performed	Not evaluated due to clinical diagnosis
P8	Mother	VS	1 yr	EEG data had movement artifacts and no conclusions could be drawn. No fMRI tests were performed	No evidence
P9^1^ & P9^2^	Father/Sibling	MCS/EMCS	1 yr	EEG revealed basic attentional capacity. No fMRI tests were performed	No evidence
P10	Father	VS	3 yrs	No significant findings with EEG. No fMRI tests were performed	No evidence
P11	Mother	VS	2 yrs	No significant findings with EEG. fMRI data had movement artifacts and no conclusions could be drawn	No evidence

VS = Vegetative State; MCS = Minimally Conscious State; EMCS = Emergence from the Minimally Conscious State; EEG = Electroencephalography; fMRI = Functional Magnetic Resonance Imaging. All clinical diagnoses are derived from repeated evaluation with the CRS-R. Main neuroimaging results column summarizes the disclosure letter provided to caregivers. * denotes neuroimaging evidence of covert consciousness. Neuroimaging results are derived from research outlined in Table 1

We conducted eleven pre-disclosure interviews and ten post-disclosure interviews between 2015 and 2016. Two family caregivers (P9^1^ and P9^2^) shared surrogate decision-making status for one patient, reducing the overall number of interviews. An additional family caregiver (P11) did not respond to requests for a post-disclosure interview. This caregiver’s pre-disclosure interview was emotionally difficult; we believe she withdrew from the study due to the emotions arising from discussing the patient’s accident. Representative quotes from participants are labeled with the participant number and “a” for pre-disclosure interview or “b” for post-disclosure interview, e.g., P1a or P1b.

Within the four above-outlined interview topics, we identified various themes across caregivers (Table 3). First, we observed that caregivers had a spectrum of expectations for the neuroimaging research, ranging from hopeful to conflicted expectations for the results. Some caregivers were glad to participate in a study that might improve the care of future patients, while others wanted to correct what they perceived as a misdiagnosis of the patient. Second, we observed that caregiver reactions to the neuroimaging evidence of preserved cognition were multifaceted. Caregivers were often pleased with this information, but some found it difficult to process and share with their extended family. Third, we observed that caregivers were either accepting or resistant to null results. Caregivers who were resistant challenged the validity of the neuroimaging methods. In some cases, caregivers believed that their personal assessments were more accurate than those used by the Owen lab. Finally, we observed that caregivers generally understood the neuroimaging research and results. Caregivers reported that, even if they didn’t understand initially, they felt comfortable interacting with the neuroimaging research team and asking questions.

**Table 3:** Summary findings

Interview topic	General finding	Representative quotes
Expectations for neuroimaging	Caregivers displayed a spectrum of expectations for the neuroimaging research, ranging from hopeful to conflicted expectations. In some cases, these expectations appeared to bear on caregivers’ acceptance of the neuroimaging results	“My hope is that this will help research understand that he wasn’t a vegetative brain, he wasn’t dead. Number one, that diagnosis was wrong, 100%. It was wrong.” (P1a) “The testing maybe would benefit [patient] in that people will have a better understanding of his brain function […] But that’s about it. I mean, it’s research, right?” (P2a)
Reactions to evidence of preserved cognition	Caregiver reactions to neuroimaging evidence of preserved cognition were multifaceted. Caregivers were often pleased with this information, but some found it difficult to process and share with others	“I’m talking to him more as an adult now. He doesn’t want to be talked to like a teenager. The more you understand, the more you know what he understands, and maybe by talking to him at a more adult level it helps him too.” (P3b) “[My in-laws equated] there was brain activity with he’s waking up tomorrow.” (P5b)
Reactions to null results	Caregivers were either accepting or resistant to null results. Caregivers who were resistant challenged the validity of the neuroimaging methods. In contrast, no caregiver who received evidence of preserved cognition challenged the results. Some caregivers also expressed emotional distress in the face of continued uncertainty	“What I do with [patient] from a sensory stimulation perspective is a lot more in-depth and aggressive, because I use acupressure therapy and muscle stimulation. They didn’t do anything like smell stimulation. I do that all the time, and I see a significant amount of reaction to that.” (P1b) “I have to know what’s going on so I could provide whatever my son needs. I just wanted to know if my son was in pain. I still feel the same. I’m lost and I’m drained.” (P10b)
Understanding of the results and study	Caregivers generally understood the neuroimaging research and results. If caregivers initially misunderstood the results, they felt comfortable asking questions of the neuroimaging research team	“They’re learning more about the brain, so it’s a matter of how people are treated. I think down the road there’s a lot of hope for different technologies. And that will change how people are treated.” (P3b) “I didn’t understand a lot, [but] when I spoke to [researcher] she broke it down and said, ‘Okay, this is what it is. There was some type of activity.’” (P5b)

Below, we describe these topics in detail. The interview topics track the experiences of caregivers as patients progressed through the neuroimaging research program. Data from other interview topics, such as caregiver experiences with health systems and caregiver burden, are reported in Munce et al. [29] and Gonzalez-Lara et al. [30], respectively.

### Caregiver expectations

Caregiver expectations for neuroimaging were complex, ranging from hope for the detection of preserved cognition to a more guarded outlook. Some caregiver expectations were shaped by a desire to correct what they perceived as diagnostic errors made by the patient’s primary care team. These disagreements often centered on a patient’s medical status. However, in some cases, caregivers also appeared to have differing beliefs about the meaning of consciousness and personhood relative to clinicians. The value of neuroimaging for these caregivers was to gain information that confirmed their beliefs and contribute to research that will mitigate these disagreements in the future. For example, one caregiver firmly believed that her spouse was misdiagnosed: “My hope is that this will help research understand that he wasn’t a vegetative brain, he wasn’t dead. Number one, that diagnosis was wrong, 100%. It was wrong.” (P1a) This patient was assessed multiple times, but no evidence of preserved cognition was observed. As we review below, lack of evidence of preserved cognition does not rule out the possibility of covert consciousness. Yet, lack of evidence could still be disappointing in light of these strong expectations.

In contrast, other participants expressed more guarded expectations. For example, one caregiver acknowledged the limits of neuroimaging when her son was examined. She expressed hope, but also the importance of being realistic: “The testing maybe would benefit [patient] in that people will have a better understanding of his brain function […] But that’s about it. I mean, it’s research, right?” (P2a) This patient was clinically diagnosed as being in the minimally conscious state, suggesting that there might be a response to neuroimaging tests. However, EEG assessment showed only sparse evidence of attentional capacities, and no conclusions could be drawn from the fMRI data.

Additionally, some caregivers’ expectations related to a patient’s cognitive functions. One caregiver described his curiosity regarding “what [his spouse] was thinking.” He wanted to know whether: “My speech, my verbal, my sound, songs, anything that different parts of the brain were responding to.” (P4a) This caregiver’s attitude could represent broader expectations that neuroimaging might justify his caregiving efforts have been beneficial. If neuroimaging revealed that his spouse retained certain perceptual capacities, he might feel justified in his decision to continue rehabilitation, or decisions in daily interactions, such as speaking or reading to her.

### Caregiver reactions to evidence of preserved cognition

“Evidence of preserved cognition” describes neuroimaging data that indicate a response to experimental stimuli, ranging from basic attentional capacities to volitional mental imagery. This evidence may suggest that patients are covertly conscious despite their clinical diagnosis. Of the patients with PDoC associated with this study, three showed definitive evidence of covert consciousness (P3, P5, P6), while three further patients showed evidence of basic attentional capacities (P2, P7, P9). Evidence of basic attentional capacities, while important for understanding the patient’s preserved cognition, is ultimately insufficient to conclude whether covert consciousness is present. In describing caregiver reactions below, we distinguish between evidence of covert consciousness and evidence of basic attentional capacities.

Caregivers who received evidence of covert consciousness reflected on the benefit of having new—and confirming—information that the patient was aware. A recurring theme was that these results might change behaviors toward the patient. For example, a father described how evidence of covert consciousness motivated him to treat his son as an adult, rather than a teenager—the age at which he sustained his brain injury: “I’m talking to him more as an adult now. He doesn’t want to be talked to like a teenager. The more you understand, the more you know what he understands, and maybe by talking to him at a more adult level it helps him too.” (P3b) This father’s observations suggest that aspects of his relationship with his son were anchored to the time of injury. Neuroimaging data appeared to beneficially disrupt these assumptions.

This father also noted how he planned to use the neuroimaging data to shape interactions with clinicians. Newer clinical staff, he observed: “Don’t know him that well, but I think [the results] are going to help how they communicate. Instead of talking to him like a baby, they’ll talk to him like a 36-year-old man, like he is.” (P3b) This father’s experience also appeared colored by the discovery of his son’s personhood and what that implied for caregivers in similar situations: “They’re people inside, right? Even though I believed that he understood all along, [the results] reinforced what I believe. Maybe I’m babying him too much, you know?” (P3b).

Another caregiver who received EEG results of attentional capacities reflected on how the data provided insight on the kinds of experiences her spouse could have, even though they did not confirm covert consciousness:

Now I know at what level I can do stuff with my husband. If I read to him or if I watch a movie with him, my thought in the past was ‘Does he understand? Does he get what I’m saying?’ But now I know he does. So, I’m like, ‘Okay, then he’s obviously in there.’ (P7b)

Notably, this caregiver’s observations highlight the utility of neuroimaging assessment in patients who are already known to be conscious. Her spouse’s clinical diagnosis, the LIS, implied that he was conscious but unable to move his body. This caregiver found value in neuroimaging data in that it revealed the kinds of perceptual experiences her spouse could have.

Caregivers who received evidence of covert consciousness also reflected on the positive feeling of having their beliefs about a patient validated. These caregivers reported dismissiveness or skepticism from clinicians, friends, and extended family when they broached the topic of preserved cognition. The neuroimaging data affirmed their beliefs and, in some cases, allowed caregivers to constructively return to these difficult conversations. For example, one mother who received evidence of covert consciousness stated: “We like to tell people who might have been a little dubious. It’s a positive feeling. People are more supportive.” (P6b).

Although most caregivers were pleased to receive evidence of preserved cognition, some expressed disappointment that the neuroimaging data fell short of explaining the cause of injury. The spouse of the patient diagnosed as being in the LIS, described above, noted that she wished the research team could have explained why her spouse’s stroke occurred. “I will never have that answer,” she stated: “When the results all came back, I was really happy. But then on the flip side I’m like, why did this happen?” (P7b). This caregiver acknowledged that the neuroimaging methods were not designed to yield this information. Nevertheless, her spouse’s brain injury left her with unanswered questions that framed her understanding of the neuroimaging research and results.

Another caregiver reflected on the difficulty of sharing the results with her children and in-laws. This caregiver was happy to receive evidence of covert consciousness, but this information was difficult to convey to others, and ultimately resulted in interfamily stress. “You always want to hear more than what you get,” she stated: “[My children] asked me questions, and I would say, ‘I don’t even know,’ and they’d question, ‘Why don’t you know, Mom?’ ‘This is Dad. How come you don’t know?’” (P5b) She noted further that her in-laws equated “there was brain activity” with “he’s waking up tomorrow,” a common misunderstanding about the prognostic value of these results. This reaction underscores how different family members might attribute varying—even conflicting—meanings to the same neuroimaging data.

### Caregiver reactions to null results

“Null results” describe neuroimaging data that are uninformative. Lack of a neural response does not imply that a patient is unconscious. A conscious patient might fail to understand the task instructions, have auditory impairments, or fall asleep during the test leading to a false negative result. As true negative and false negative results are indistinguishable, the neuroimaging research team framed these results as “null” or “uninformative” during disclosure.

Caregivers who accepted null results appeared to do so for two reasons. First, some appeared to have come to terms with a patient’s condition. For example, one caregiver of a patient who was clinically diagnosed as being in a VS (P4b) said that he “knew from day one” that he’d likely receive null results. It is unclear whether this reaction was related to the length of time the patient was in a VS. This caregiver’s spouse was in a VS for 1.2 years, while other caregivers whose spouses or children had been in a PDoC for much longer were ultimately resistant to null results.

A second reason why caregivers appeared accepting of null results is that they understood potential technical failures in the neuroimaging process. One caregiver stated, for instance: “I had a chat with [the research team], just before Christmas, and as I suspected the results are very inconclusive because [patient] moved too much during the MRI. It wasn’t a big, ‘Oh, my goodness’ surprise, to be honest. But anyway, it’s been worth a go.” (P2b).

In contrast to caregivers who were resigned to null results, others refused to accept the results and challenged the validity of the neuroimaging tests. One caregiver, who was adamant that her spouse was responsive at home, was quick to dismiss the tests as mere “research” interventions. She stated: “What they do in research is not final, right?” (P1b) This caregiver continued to challenge the validity of neuroimaging, arguing that the sensory stimulation she performs is more sensitive than the tests used in the Owen lab. For this caregiver, neuroimaging did not compare with the daily attention she provided to the patient. She observed further:

What I do with [patient] from a sensory stimulation perspective is a lot more in-depth and aggressive, because I use acupressure therapy and muscle stimulation. They didn’t do anything like smell stimulation. I do that all the time, and I see a significant amount of reaction to that. (P1b)

She also expressed disappointment at not receiving more information about alternative research interventions: “I asked about transcranial stimulation, I asked about different therapies that could help with brain activity. Not that I should expect anything, but I thought somebody in that field might have an idea of other kinds of therapy that could be tried.” (P1b) Interestingly, this caregiver’s observation does not accurately reflect how the Owen lab discusses research results. During disclosure, it is common to provide evidence-based recommendations or to invite caregivers to enroll patients in future studies. However, the research team carefully avoids discussion of unproven interventions. At the time of disclosure, there was no evidence that transcranial stimulation would benefit this patient.

Finally, some caregivers’ reactions revealed emotional exhaustion in the face of continuing uncertainty. The father of one patient who received null results was accepting of them, but ultimately expressed disappointment in not knowing if his son was in pain: “I have to know what’s going on so I could provide whatever my son needs. I just wanted to know if my son was in pain. I still feel the same. I’m lost and I’m drained.” (P10b).

### Caregiver understanding of the study and results

Overall, caregivers appeared to understand the neuroimaging research program and the results. One caregiver acknowledged that, although she didn’t understand the “highly technical stuff,” she still “understood enough to feel very comfortable with what was going on” (P6b). Caregivers also understood the potential benefit of the neuroimaging research. A father, whose son had been in a PDoC for 17 years, stated: “They’re learning more about the brain, so it’s a matter of how people are treated. I think down the road there’s a lot of hope for different technologies. And that will change how people are treated.” (P3b).

Although caregivers appeared to generally understand the neuroimaging research, we observed variation in how they described and attributed meaning to the results. A key theme among several caregivers was the use of the phrases “there is activity” and “brain activation.” For example, one caregiver recalled her experience of hearing the word “activity” during the disclosure consultation. She stated: “All I had in my mind was, ‘Okay, there’s activity.’ And a lot of things got blocked out. I didn’t want to hear anything else.” (P5b) Notably, the information this caregiver “blocked out” and “didn’t want to hear” pertained to technical features of the neuroimaging tests. The term “activity” was the focal point of her understanding.

One caregiver also stated that she wished she knew more about the experimental stimulus. This caregiver’s son underwent a naturalistic stimuli test (see Table 1, Naci et al. 2014). The test used a short audio clip from the movie, Taken, to evoke a neural marker of executive processing and suspense. The film was discussed during the informed consent process, however the caregiver was unfamiliar with it:

I would have liked to have been able to hear what [patient] was hearing. We weren’t familiar with the [movie ‘Taken’]. My husband Googled it later and found that it was a very violent movie with profanity. [Patient] didn’t live a sheltered life, but I thought if it was being traumatic to him emotionally, if it was really violent or vulgar or there were things that were really agitating him inside, how would we know if we’re not hearing? (P8b)

The neuroimaging data from this patient contained movement artifacts, potentially from agitation while the patient was in the scanner. Although the audio clip used in the study does not contain profanity or violence, the caregiver might have reasonably thought that the stimulus caused this agitation.

While there was variation in caregiver understanding of the results, many were quick to contextualize their beliefs in light of positive experiences with the neuroimaging research team. Caregivers reported feeling neglected by the health system; they believed that patients with PDoC were not treated as persons and received suboptimal care. In contrast, caregivers reported that the neuroimaging research team treated patients with respect. Indeed, one caregiver who received null results recalled the interaction with a research team member:

He said, ‘I just want to prepare you all in advance this is only a research test.’ And I turned around and I looked at him and I said, ‘You know what, Doctor? Just the fact that you have come all the way to do the test, that’s all I can be thankful for. Doesn’t matter what the results are.’ (P1b)

Additionally, caregivers also highlighted the neuroimaging research team’s systematic approach to discussing the results. This appears to have tempered expectations and increased comprehension. One caregiver stated: “I didn’t understand a lot, [but] when I spoke to [researcher] she broke it down and said, ‘Okay, this is what it is. There was some type of activity.’” (P5b) Similarly, the caregiver who expressed concern about the test intervention, described above, still praised the neuroimaging research team: **“**I was very impressed with [researcher]. He was very easy to talk to, you know what I mean? He wasn’t gruff and cold, he just seemed very warm and personable, so that was positive.” (P8b) The neuroimaging research team’s efforts to earn trust, rather than expect it, appears to have enhanced their relationship with caregivers and the quality of the disclosure process.

## Discussion

This study reports, for the first time, preliminary insight on the expectations and experiences of caregivers of patients with PDoC when they receive neuroimaging results of covert consciousness in the research context. Overall, caregivers appeared to understand the neuroimaging research, had positive experiences with the neuroimaging research team, and found the results valuable. These findings cast doubt on speculative ethical concerns regarding therapeutic misconception and false hope. While it is possible that therapeutic misconception or false hope could arise from the disclosure of complex neuroimaging data following severe brain injury, our study did not detect these attitudes among our participants.

Although our findings contradict these cautionary ethical arguments, we did observe complexities in caregiver expectations, the meaning attributed to neuroimaging results, and the effect of interactions with the neuroimaging research team. One critical issue was the potential for disagreement between caregivers and researchers regarding the neuroimaging results. Some caregivers who received null results disagreed with the findings and the validity of the neuroimaging tests. In contrast, no caregiver who received results of covert consciousness disagreed with the findings. Previous qualitative research details similar findings. Schembs et al. observed that, when next-of-kin disagree with EEG results acquired from a patient with PDoC, they might also dismiss the validity of the neuroimaging test [17, 18]. Crucially, Schembs et al. also observed that caregiver disagreement was linked to receipt of null results, not evidence of covert consciousness.

Additionally, we also observed that some caregivers approached the neuroimaging research through the lens of their own lay vocabulary or conceptual framework. Lay vocabularies and conceptual frameworks related to brain injury did not appear to impede caregiver understanding of the results, in part, due to ongoing counseling provided by the neuroimaging research team. However, previous qualitative research has identified this as a potential contributor to communication breakdown. Edgar et al. observed that clinicians use “a medical science framework” to describe the status of patients with PDoC, which constructs “the patient in terms of measurable physical parameters.” Family members, by contrast, use an “interpretative framework that encompasses the uniqueness of the patient and the relative’s relationship to them” [31]. Failure to recognize this difference can lead to “pathologizing” interactions with caregivers [32].

We did not observe these negative interactions. However, we acknowledge that there could be discrepancies in the ways that caregivers and health professionals understand—or misunderstand—the neuroimaging data upon disclosure. First, caregivers might fail to understand the neuroimaging intervention (or the experimental design). One caregiver in our study (P8b), for example, explained that she wished she knew more about the auditory stimulus from the film, Taken. This caregiver’s misunderstanding was eventually corrected with further information, but the discrepancy highlights potential failures in the informed consent process.

Second, caregivers might fail to understand the rigor and validity of the neuroimaging tests. This misunderstanding could emerge alongside strong expectations for evidence of covert consciousness (e.g., in our study, P1b). These caregivers likely understand the neuroimaging procedure, the results, and their potential clinical value. Yet they may ultimately misunderstand the degree of certainty that should be placed on the neuroimaging data.

Third, caregivers might fail to understand the diagnostic or prognostic utility of the neuroimaging data. In our study, for instance, one caregiver (P5b) reflected on how her in-laws—the patient’s parents—equated observed brain activity with recovery. The caregiver’s in-laws ultimately reasoned “beyond the evidence” by attributing greater prognostic value to the neuroimaging data. Importantly, the caregiver’s in-laws were one step removed from the disclosure process, and so their understanding of the results was out of the control of the neuroimaging research team. Nonetheless, this kind of family dynamic might still indirectly impact clinical decision making, and health professionals should be prepared for these situations.

Finally, health professionals themselves might also misunderstand caregivers’ conceptualizations of the neuroimaging results and associated goals of care. As described above, a caregiver’s understanding of a patient’s conditions is often embedded in ongoing and complex familial relationships. This understanding, however, might not cohere with received medical wisdom. Failure of health professionals to acknowledge this alternative way of understanding a patient’s condition can undermine trust with caregivers, and potentially interfere with clinical decision making for the patient.

In light of these variations in understanding neuroimaging evidence of covert consciousness, we outline below two steps that might improve the disclosure process in both research and clinical settings. First, prior to disclosing neuroimaging results, professionals should endeavor to understand caregiver values and the language they use to describe a patient’s condition. These pre-disclosure discussions might prevent misunderstandings between caregivers and professionals [33]. Professionals needn’t acquiesce to caregiver beliefs about patients with PDoC that are inconsistent with scientific facts. However, acknowledging caregiver views could lead to better communication. Updated clinical guidelines on PDoC explicitly recommend that physicians familiarize themselves with patient and family values to improve counseling on high-stakes decisions [12].

Second, disclosure of neuroimaging results to caregivers should be standardized, involve professionals who are trained in communication, and allow sufficient time for questions and discussion [10, 34, 35]. These mechanisms are intended to mitigate disagreement, improve caregiver comprehension of the results, and potentially optimize decision making.

Standardized disclosure procedures might be adapted from cognate fields, such as Alzheimer’s disease (AD) research. Disclosure standards for PET imaging data suggestive of a pre-clinical AD diagnosis could be instructive. Harkins et al. developed a method for disclosing these results to mitigate detrimental emotional responses and caregiver/patient misunderstanding [36]. This procedure involves pre- and post-disclosure educational interventions to temper expectations. Patients and caregivers are also assessed for the impact of disclosure on their wellbeing and willingness to learn new information. There are relevant disanalogies between the disclosure of a pre-clinical AD diagnosis and evidence of covert consciousness following severe brain injury. However, the procedure for standardizing disclosure of neuroimaging results could be a first step for the PDoC research community.

## Limitations

Our study has several limitations. First, although our study reached thematic saturation, only three patients cared for by interview participants displayed evidence of covert consciousness. This may have impacted the variation in our observations of caregiver reactions to neuroimaging results. Moreover, we were unable to compare these reactions across caregiver types. Caregiver age, relationship to patient, self-reported gender, and duration of condition might impact their expectations and understanding.

Second, caregivers who seek out assessment at the Owen Lab are often familiar with the lab’s research from popular press coverage. This could bias caregiver understanding of neuroimaging results or lead to unrealistic expectations. We did not observe this in our study, but biases could be undetected.

Third, we interviewed caregivers of patients with PDoC who underwent a battery of neuroimaging tests, not a single test. Specific tests were not used in some patients due to agitation, metal implants, or the inability to be in a prone position for a scan. We could not assess caregiver attitudes toward particular neuroimaging tests.

## Conclusion

This study reports, for the first time, the expectations and experiences of caregivers of patients with PDoC as they undergo neuroimaging research to detect covert consciousness. Overall, caregivers appeared to understand the neuroimaging research and results, casting doubt on speculative ethical concerns regarding therapeutic misconception and false hope. Nonetheless, we observed that some caregiver expectations deviated from the neuroimaging results, raising the potential for disagreement. We identified two avenues that could improve the disclosure process: supporting trust-building interactions among caregivers, clinicians, and researchers, and standardizing disclosure procedures. Attention to the experiences of caregivers, their understanding of neuroimaging, and how it is mediated by their interactions with researchers and clinicians, could lead to improved communication of neuroimaging results following severe brain injury.

## Supplementary Information

**Additional file 1.** Sample disclosure letter.

**Additional file 2.** Interview guide.

## Data Availability

The datasets generated and/or analyzed during the current study are not publicly available to protect participant confidentiality but are available from the corresponding author on reasonable request.

## Abbreviations

PDoC: Prolonged disorder of consciousness
EEG: Electroencephalography
fMRI: Functional magnetic resonance imaging
PET: Positron emissions tomography
VS: Vegetative state
MCS: Minimally conscious state
EMCS: Emergent from minimally conscious state
ICU: Intensive care unit
