# Supplementary material for: Caregiver reactions to neuroimaging evidence of covert consciousness in patients with severe brain injury: a qualitative interview study
Source: BMC Med Ethics. 2021 Jul 28;22:105. doi: 10.1186/s12910-021-00674-8 (PMC8320067; doi:10.1186/s12910-021-00674-8)
Supplement: Supplementary file 1 — Additional file 1. Sample disclosure letter. [file 12910_2021_674_MOESM1_ESM.pdf]

Impact of neuroimaging on the families of vegetative and minimally conscious patients after serious brain injury

Principal investigator: Charles Weijer
November 17, 2013

Semi-structured interview guide: first interview

Please note that this guide only represents the main themes to be discussed with the participants and as such does not include all the various probes that may be used. Non-leading prompts will also be used, such as "Can you please tell me a little bit more about that?" and "What does that look like for you" when probing abstract claims or statements.

Warm-up and establishment of rapport

Thank you for agreeing to participate in this interview. We are interviewing you to better understand your experiences as the decision maker for a person who is participating in a research study. So, there are no right or wrong answers to any of our questions. We are interested in your opinions.

You have signed the consent form and are aware of your rights as a participant. The interview should take approximately one to one-and-a-half hours depending on how much information you would like to share. With your permission, I would like to audio record the interview because I don't want to miss any of your comments. All responses will be kept confidential. This means that your de-identified interview responses will only be shared with research team members and we will ensure that any information we include in our report does not identify you to anyone. You may decline to answer any particular question or stop participating in the interview altogether at any time and for any reason. Are there any questions about what I have just explained?

May I turn on the digital recorder?

Relationship with patient

I'd like to start by asking you to share with me your relationship with (name). I understand that (name) is your (relation). Who would you describe as his/her primary care giver?
Prompts: Are there any other family members or friends involved in his/her care? Who makes decisions on behalf of (name)? How do you feel about that?

If not self-identified as primary caregiver: What do you understand to be your primary role in relation to (name)?

Do you also have other roles (i.e., family member, care giver, and decision maker)? How do you manage these various roles? Can you give me a concrete example of a time when you felt your roles were in conflict?

Patient's current medical condition

Now I'd like to talk to you a little bit about what's happened with (name). Can you tell me a little bit about that?
Prompts: What happened to him/her? How long has he/she been here (in a hospital or at home)? Where would you like him/her to be?

Could you please describe for me your understanding of (name)'s medical condition? How do you explain it others in your family for instance?
Prompts: What is it called? What does it mean in terms of the current life of (name)? What are the prospects for recovery?

Relationship with care providers

Where do you get information about (name)'s medical condition?
Prompts: Nurses? Physicians? Neurologists? Family members or friends? Others?

How do you figure out whether a source of information is reliable?
Prompts: For example, have you ever had difficulty choosing between conflicting opinions?

Can you tell me about one of the most helpful experiences you've had with a care provider?

And what about one of the least helpful experiences?

Who do you rely on the most for support? What type of support do you feel you need?
Prompts: Nursing help? Social worker? Family? Friends?

Key decision-making moments

Can you tell me about a recent decision that needed to be made regarding his/her care?
Prompts: What happened? Who was involved?

What for you were the key decisions that needed to be made?
Prompts: How did the topic come up? How did you feel about that discussion? What was the outcome?

Experiences of being with the patient

Now I want to ask you some questions about your experiences of being with (name). How would you describe your experience of (name) when you are with him/her?

I'd like to talk to you about your experiences of (name) knowing when you are in the room—what do you think about this? What about his/her ability to feel pain or discomfort? Or when you hold his/her hand? How does that make you feel?

What are your thoughts about whether or not he/she communicates with you?

How does he/she do this? What was going on that made you believe this (grimaced, responded to touch, etc)?

What about how you communicate with him/her? For example, sometimes people talk to their family members. What is that like for you? What do you think about his/her ability to hear you?

How do others in your life react when you express your thoughts about this? Does anyone share your view? Does anyone disagree (family members, physicians, nurses)?

Reasons for participating in research

How did you come to the program of research on serious brain injury at Western?
Prompts: Did someone recommend that (name) undergo testing here? If so, what did he/she tell you about the sort of testing we do here?

What made you interested in having him/her participate in the study?
Prompts: Do you think testing will benefit (name)? How? What do you hope to learn? Are you hoping for anything to change (i.e., future care, medical condition)?

Are there any risks to (name) of being in this study? What are they?

Are there any risks to you or anyone else in the family of (name) being in this study? What are they?

Do you think his/her medical condition will improve? What do you think it will be like in 1 year? 5 years? Do the others in your family share this view?

Close

Thank you very much for speaking with me today. Is there anything else I haven't asked you about that you would like to share with me?

Before we end I want to explain to you what our next steps will be in this study. We will send you a summary of our interview today about a week before our next meeting and then sit down and speak with you again after your participation in the program of research on serious brain injury at Western University.

Semi-structured interview guide: second interview

**Families will have been provided a 1 to 2 page summary of the first interview.*

Please note that this guide only represents the main themes to be discussed with the participants and as such does not include all the various probes that may be used. Non-leading prompts will also be used, such as "Can you please tell me a little bit more about that?" and "What does that look like for you" when probing abstract claims or statements.

Warm-up and establishment of rapport

Thank you for agreeing to speak with us again. As you know, we are interviewing you to better understand your experiences as the decision maker for a person who is participating in a research study. So, there are no right or wrong answers to any of our questions. We are interested in your opinions.

You have signed the consent form and are aware of your rights as a participant. The interview should take approximately one to one-and-a-half hours depending on how much information you would like to share. With your permission, I would like to audio record the interview because I don't want to miss any of your comments. All responses will be kept confidential. This means that your de-identified interview responses will only be shared with research team members and we will ensure that any information we include in our report does not identify you to anyone. You may decline to answer any particular question or stop participating in the interview altogether at any time and for any reason. Are there any questions about what I have just explained?

May I turn on the digital recorder?

Reflections on first interview

I'd like to start by asking you your thoughts about our first conversation.

What was it like to read the summary?

How well did the summary capture the main points of what you remember of our conversation?

Often people remember things they might have wanted to share after a conversation is over. Is there anything else that was left out of that first conversation that you think is important for us to know?

Experiences of participation in study

Since we last met, you went on to participate in the program of research on serious brain injury at Western University. What was it like to participate in that study?

How did you feel you were treated by the study staff?

How did you feel during the process (anxious/relaxed/neutral)?

Please tell me about anything that you didn't expect.
Prompts: in terms of the study results; in terms of how you felt; in terms of the reactions of others

---

Study Results

What was it like to receive the study results?
Prompts: Who explained the results to you? Where were you? Who was with you?

What do you remember about what was explained to you?

Was there anything you wanted to know and weren't told?

Was there anything you felt you didn't understand?

Overall, how did you feel about the study results?

What did you do right after receiving the results?

Impact on beliefs

Since receiving the study results, how would you describe your experience of (name) when you are with him/her?

I'd like to talk to you about your experiences of (name) knowing when you are in the room—what do you think about this? What about his/her ability to feel pain or discomfort? Or when you hold his/her hand? How does that make you feel?

What are your thoughts about whether or not he/she communicates with you?

How does he/she do this? What was going on that made you believe this (grimaced, responded to touch, etc)?

What about how you communicate with him/her? For example, sometimes people talk to their family members. What is that like for you? What do you think about his/her ability to hear you?

How do others in your life react when you express your thoughts about this? Does anyone share your view? Does anyone disagree (family members, physicians, nurses)?

Recommendations to other families

What advice would you give to other families considering participating in this research?

Finally, thinking back on your experiences, what or who has most helped you to get through this experience? What could have made it better?

Close

Thank you so much again for your valuable time in speaking with us. It is truly appreciated.
