# Supplementary material for: Caregiver reactions to neuroimaging evidence of covert consciousness in patients with severe brain injury: a qualitative interview study
Source: BMC Med Ethics. 2021 Jul 28;22:105. doi: 10.1186/s12910-021-00674-8 (PMC8320067; doi:10.1186/s12910-021-00674-8)
Supplement: Supplementary file 2 — Additional file 2. Interview guide. [file 12910_2021_674_MOESM2_ESM.pdf]

<u>Sample disclosure letter</u>

REFERING PHYSICIAN
ADDRESS

Re: PATIENT

Dear Dr. XX,

I am writing to provide you with some feedback about Mr. YY, who was included as part of our research study this year. We first visited and assessed
him at HOME/FACILITY in DATE, and the results suggested that he would be a suitable candidate for bringing to London, Ontario. A four-day visit was arranged for a more extensive assessment in DATE. The summary results are as follows:

Behaviour:
Mr. YY was assessed with the Coma Recovery Scale – Revised (Giacino et al., 2004) during our first visit and N additional times during his admission in London. His diagnosis based on all of these assessments was 'Vegetative State' and on no occasion did Mr. YY show any behavioural evidence of command following.

Electrophysiology:
A variety of electroencephalography (EEG) and event-related potential (ERP) tasks were attempted with Mr. YY, and are described below. EEG/ERP uses small electrodes placed on the scalp to record the electrical signals generated by the cortex. When an area of the brain beneath an electrode is active, it will produce specific electrical patterns. By recording these electrical signals, we can compare brain activity in response to different types of stimuli, in order to determine how and when the brain is responding appropriately.
In Mr. YY's case, we were only able to acquire good quality data during the following EEG/ERP tasks:

1. <u>Language Processing</u>: There was statistically significant evidence for ERP differences between speech and non-speech sounds, indicating the preservation of (at least) the ability to perceive speech. Whether this processing also indicates comprehension of the words is unclear.

2. <u>Command-following</u>: There was no evidence that the brain was responding appropriately to instructions to move, or to imagine moving (e.g. the hands or feet). This is driven partially because there were certain artefacts from muscle movements, and other normal physiological processes, including sleep, so that it was difficult to obtain a sufficient amount of EEG data to analyze. It does not necessarily mean that Mr. YY was not able to perform the task, or that he was not trying to perform the task, but nevertheless, we were not able to detect a reliable EEG response to indicate that he was following the instructions.

In conclusion, from the electrophysiology, we can conclude that Mr. YY's brain was able to differentiate speech from other types of sounds, although it is unclear to what extent he may have comprehended the meaning of the words. There was no reliable evidence that he was able to follow commands to move or imagine moving. However, it is important to stress that these are 'null' results and should be interpreted with some caution (see further explanation below in relation to fMRI findings).

fMRI

Mr. YY underwent a set of tests using functional magnetic resonance imaging (fMRI). This technique is used to observe, in a non-invasive fashion, changes in the brain's blood flow to various kinds of stimulation (e.g. visual stimulation, auditory stimulation). When a specific part of the brain is engaged by some type of stimulation, it will require increased blood flow, which appears, in our data, as red/yellow 'blobs'.

We performed the following fMRI tests:

1. <u>Movie</u>: To examine the brain's responses to detailed visual and auditory information, we played a short (8 minutes) movie. We found evidence of basic sensory processing in the auditory domain. In particular, we observed very strong responses in the auditory cortex that were indistinguishable from healthy controls, suggesting basic sound processing. In addition, we found responses to language in the same regions as controls, which suggested speech processing. We did not observe any responses in the occipital cortex, suggesting a lack of basic visual processing. Similarly, we observed no evidence of executive function related to the movie content. This may, in part, be explained by Mr. YY's absence of visual processing, which may have hindered his understanding of the movie.

2. <u>Attention – Command-following</u>: To examine more specifically Mr. YY's ability to pay attention, we played sounds, and asked him to either *'Count'* (the number of times he heard a word e.g., 'yes') or '*Relax*' (do nothing at this time), for 5 minutes. We found increased activity for words that were to be attended, than for words that were to be ignored. This result suggested that Mr. YY was able to pay attention and follow commands to perform the task.

3. <u>Attention – Communication</u>: We also asked Mr. YY simple questions that had a 'yes' or 'no' answer, such as: "Are you in a hospital?" or "Is your name YY?". We found evidence that Mr. YY was able to attend selectively to one of the two words more than the other ("yes/"no"), so as to convey the correct answers to two of the four questions asked.

4. <u>Command-following:</u> Mr. YY was asked to perform two mental imagery tasks, which involved imagining playing tennis (motor imagery) and imagining moving around the rooms of his home (spatial navigation), whilst he was scanned. Unfortunately, no reproducible or sustained areas of activation, like those that are typically observed in healthy volunteers when performing this task, were observed on repeated assessment.

In conclusion, in our opinion, the fMRI data is consistent with the EEG/ERP data in suggesting a higher level of awareness than can be deduced based on behaviour alone. The fMRI data provided evidence of the ability to understand the requirements of a simple command following task (e.g. "count the number of time you hear a word") and there is evidence of the ability to attend to specific types of information.

Because we observed that during some sessions MR. YY appeared more tired than at others we would appreciate the opportunity to follow up with him in the future.

If you require further information, please do not hesitate to contact me.

Adrian M. Owen
Canada Excellence Research Chair in Cognitive Neuroscience and Imaging
Brain and Mind Institute, Room 224
Natural Sciences Centre
Western University
London, Ontario N6A 5B7
